# Who benefits from free institutional delivery? evidence from a cross sectional survey of North Central and Southwestern Nigeria

**DOI:** 10.1186/s12913-017-2560-1

**Published:** 2017-09-02

**Authors:** Anthony I. Ajayi, Wilson Akpan

**Affiliations:** 1 Department of Sociology, Faculty of Social Sciences & Humanities, University of Fort Hare, East London, South Africa; 2 Faculty of Social Sciences & Humanities, University of Fort Hare, East London, South Africa

**Keywords:** Maternal health, Free maternal health policy, User-fee removal, Inequality

## Abstract

**Background:**

The reasons for low utilisation of maternal health services in settings where the user-fee removal policy has been implemented continue to generate scholarly debates. Evidence of whether user-fee removal benefits the poor women in underserved settings is scanty and inconsistent. This article examines use of maternal health care services in the context of free maternal healthcare and profiles the beneficiaries of user-fee removal.

**Methods:**

The study adopted a descriptive design. A three-stage cluster sampling method was used to select a representative sample of 1227 women who gave birth between 2011 and 2015. Questionnaires were administered using a face-to-face interview approach and data generated were analysed using descriptive and inferential statistics.

**Results:**

The analysis shows that the use of maternal healthcare services has improved considerably in North Central and Southwestern Nigeria. While socioeconomic and geographical inequality in the use of maternal healthcare services appear to be disappearing in Southwestern Nigeria, it appears to be widening in North Central Nigeria. The findings indicate that 33.6% of women reported to have benefitted from the free child-delivery programme; however, substantial variation exists across the two regions. The proportion of beneficiaries of user-fee removal policy was highest in urban areas (35.9%), among women belonging to the middle income category (38.3%), among women who gave birth in primary health centres (63.1%) and among women who resided in communities where there was availability of health facilities (37.2%).

**Conclusion:**

The study concludes that low coverage of the free maternal health programme, especially among women of low socioeconomic status residing in underserved settings is among the reasons for persistent poor maternal health outcomes in the context of free maternal healthcare. A model towards improving maternal health in underserved settings, especially in North Central Nigeria, would entail provisioning of health facilities as well as focusing on implementing equitable maternal health policies.

## Background

Socioeconomic and geographical inequalities in the use of maternal health care services in many developing countries are well documented [1–3]. Available evidence shows that inequality in access to maternal healthcare services remains persistent despite scaling up of maternal health interventions in low and middle-income countries [1, 4]. Previous studies suggest that user-fee removal for maternal healthcare is linked with an increase in the use of skilled birth facilities [5–8]. However, despite the introduction of user-fee removal for maternal healthcare services, maternal health outcomes of women in the lowest wealth index remain persistently poor [1, 4]. The reasons for this are not well understood and continue to generate scholarly debates. Distance, lack of transportation, poverty, and poor quality maternal health services were among the common explanations advanced for poor maternal outcomes despite scaling up of maternal health interventions in many developing countries [9–11]. However, these reasons do not sufficiently explain why maternal health outcomes of women in the lowest wealth index remain persistently poor. Consequently, some scholars have begun to express doubts about whether the removal of user fees for maternal health guarantees universal access to maternal health care services [12, 13].

The central argument of this article is that persistent poor maternal health of poor women in the context of free maternal healthcare services is due to inequity in the allocation of maternal health interventions, especially free maternal healthcare intervention. A study that reported that public spending on maternal health (including foreign aids) is concentrated on the more affluent population rather than on the indigent population [13] lends credence to this argument and at least provides grounds for further investigation.

Evidence of the impact of user fees exemption for maternal health on inequality in access to maternal health is scanty and inconsistent. There is evidence that free maternal healthcare reduces [9, 14], widens [15–18], and fail to impacts [5, 10, 19] inequality in access to maternal healthcare services. While one study reported that the rate of increase in the use of maternal healthcare services was highest among women in the lowest income strata following the removal of user fees [9], other studies provide contradictory evidence and even assert that free maternal health intervention benefits women in high and middle income categories more than low-income earners [10, 18, 19]. The inconsistency of the findings pertaining to the impact of free maternal healthcare may, however, be due to differences in implementation of free maternal healthcare in different settings.

The main thrust of this article is on whether “need rather than privilege” [11] was considered in the allocation of maternal health interventions. Focusing on Nigeria—widely regarded as one of the countries with the highest burden of maternal deaths [20] —this study drew data from a cross-sectional survey to examine maternal outcomes in the context of free maternal healthcare. The study also estimates the coverage of free maternal healthcare intervention and examines the profiles of beneficiaries.

A substantial social inequality gap exists in the use of facility-based antenatal care, child delivery care and postnatal care in Nigeria [21]. The Nigeria Demographical and Health Survey (NDHS) report shows massive rural/urban disparities in the use of essential maternal healthcare services for childbirth (22% of women residing in rural areas compared to 61.7% of women in urban areas) and antenatal care (53.3% in rural areas compared to 89.4 in urban areas) [21]. There is also a notable inequality gap by level of education (11.7% with no formal education compared to 93.2% with more than secondary education) and wealth index (5.7% in women of lowest wealth index compared to 85.3% in women of highest wealth index) [21].

Nigeria has witnessed a relatively substantial expansion of maternal health interventions [22–26]. In 2012, a national free maternal and child healthcare programme was initiated (a component of the subsidy reinvestment programme) to complement the already existing Midwife Service Scheme programme initiated in 2009. Besides these programmes, many federating states initiated various maternal health programmes to complement the national free maternal health programme. At best, need-based allocation of the funds injected to maternal health in the past few years should substantially improve maternal outcomes. However, the key question remains whether these interventions reach women with unmet needs for maternal health services or women in underserved communities.

## Methods

The data analysed here are derived from a larger project investigating maternal outcomes in the context of free maternal healthcare, maternal narratives about free maternal healthcare, and users’ experiences of free maternal healthcare in Southwestern and North Central Nigeria (MACONFREE Study). The survey data of 1227 women who gave birth in 5 years preceding the survey from the earlier study were analysed to estimate the coverage of free maternal healthcare intervention and profile its beneficiaries. Overall, the data collection took place in 20 rural, 13 semi-urban, and 10 urban areas. The study took place in Nasarawa State in North Central, Ondo State and Ekiti State in Southwestern Nigeria, which are two of Nigeria’s six main geopolitical zones. Three of the 12 states in the two regions that met the selection criteria were purposively selected. To qualify for selection a state must have had free maternal healthcare in operation during the 5 years the study was reviewing. However, two states were selected in the Southwestern region due to parallels observed in the free maternal healthcare policy in these states. While the universal free maternal healthcare programme was initiated in Ondo State in 2010, this was not the case in Ekiti State, which opted for partial fee-removal over the period. Besides this, Ondo State has contrasting maternal outcomes compared to other states in the Southwestern region [21]. However, one state was selected in the North Central region due to the similarity in the free maternal healthcare programme in the states in the region.

### Study design and sampling

This cross-sectional evaluation study was carried out between May and September 2016. Structured questionnaires were administered to 1227 women (in 1227 households) within reproductive age (15-49 years) who gave birth in the 5 years preceding the survey (2011-2015). A three-stage cluster sampling method was used to select a representative sample of women in each of the three states included in this study. Each state was clustered into enumeration units and stratified based on rural areas, towns and cities. Simple random sampling was used to select Enumeration Areas (EAs) from the list of EAs in the 2006 census, with probability proportional to size. Approximately 25 clusters per state were required to achieve the sample size. In each enumeration unit, 15 to 30 households were randomly selected in each enumeration unit. To match the NDHS cluster household survey design and its calculated sample size design effect, at least 15 eligible women were interviewed in each EA. A sample size of 409 was estimated for each state after adjusting for possible incomplete data. The sample size calculation was done using sample size calculator [27] and at a confidence level of 95%, confidence interval of ±5, and using an infinite population. The sample size was distributed equally to each state; hence, 409 participants were selected from each state. This is important in order for the derived results to be representative of each state and also to enable us to draw a valid conclusion on each state. The study took place in a total of 81 EAs. Every 10th household in each enumeration area was visited to identify study participants until the sample size of 1227 women was reached. Households without women who gave birth during the specified period were skipped; and only one woman was selected in a household irrespective of the number of “eligible” women there.

### Instrument and measurements

A pretested questionnaire measuring maternal health indicators used for the NDHS 2013 survey was administered to 1227 women across the study settings. Research assistants, who were fluent in the participants’ local language, were recruited and trained by the researchers. Interviewers approached women with respect and sought informed consent from each woman before completing the questionnaire. On average, an interview took 25 min. Women were interviewed using a questionnaire comprising three main sections: demographic characteristics (including age, and education level) household economy and socio-economic information, and ownership of goods. These questions were similar to those in the NDHS 2013 questionnaire to enable comparisons of maternal health indicators. The second section consisted of questions probing the use of maternal healthcare services. The last part contained questions probing the beneficiaries’ experiences of free maternal healthcare. Questions included summary information on quality of care and satisfaction.

### Baseline results

Data from the 2008 and 2013 Nigeria demographical and health survey (NHDS) [21] were obtained to serve as a baseline for later comparison to the study’s findings. Specifically, the rate of utilisation of skilled birth facilities for antenatal and child delivery care reported in the two surveys was compared to the proportion reported in this study. The NDHS 2008 report shows that the proportion of women that sought antenatal care in a skilled birth facility was 91.3% in Ekiti State, 77.6% in Ondo State, and 87.6% in Nasarawa State. There was, however, a notable reduction in this proportion in 2013 in Ekiti State (86.8%) and Nasarawa State (63.2%). There was slight improvement in Ondo State with an increase of about 1 %. Further, according to the NDHS 2008 report, the proportion of births that took place in health facilities in Ekiti State was 80.0%, 57.0% in Ondo State and 29.8% in Nasarawa. However, the 2013 NDHS report shows that skilled birth attendants assisted 84.7% of births in Ekiti State. Skilled birth attendants assisted only 40.7% of births in Nasarawa State compared to 67.2% of births in Ondo State.

### Statistical analysis

Obtained data were captured and analysed with the aid of Statistical Package for the Social Sciences (SPSS version 21). Descriptive analyses and frequencies were run for all variables of interest. The outcome variables (use of skilled birth facilities for child delivery) were cross-tabulated with participants’ background characteristics and a *p*-value less than 0.05 was used to determine variables that were significantly associated with the outcome variables.

### Ethical approval

The University of Fort Hare’s Research Ethics Committee (UREC) approved the study protocol (AKP031SAJA01). Written consent to participate was obtained from all study participants after explaining the aim of the study, and they alluded to understanding the aim of the study. The study adhered to the ethical principles of voluntary participation, right to privacy, anonymity and confidentiality.

## Results

### Sociodemographic characteristics of study participants

All participants in the study had given birth to at least one child, and the highest number of children given birth to by one woman was 13. Overall, participants had 3348 children with 1815 births occurring between 2011 and 2015. The average number of children per participant was 2.8 ± 1.5 children. The average age of the participants was 30.4 ± 6.3 years. As shown in Table 1, the majority of the participants were married (95.9%), Christians (76.9%), Yoruba (59%), owned a mobile phone (89.1%), watched television regularly (91.6%), had formal education (92.3%), and reported earning an income (70%). However, only half of the participants owned a bank account and even less used the internet (27.9%).

**Table 1:** Demographic characteristic of the respondents by study areas

Variables	Overall *N* (%)	Ekiti *n* (%)	Ondo *n* (%)	Nasarawa *n* (%)
Age Groups				
20 and below	69 (5.7)	15 (3.8)	13 (3.2)	41 (10.0)
21-25	239 (19.8)	62 (15.5)	64 (15.9)	113 (27.6)
26-30	368 (30.4)	125 (31.3)	121 (30.1)	122 (29.8)
31-35	276 (22.8)	95 (23.8)	103 (25.6)	78 (19.1)
36-40	189 (15.6)	75 (18.8)	70 (17.4)	44 (10.8)
40 and above	69 (5.7)	27 (6.8)	31 (7.7)	11 (2.7)
Marital Status				
Currently married	1163 (95.9)	374 (93.5)	398 (99.0)	391 (95.1)
Formerly Married	12 (1.0)	4 (1.0)	1 (0.2)	7 (1.7)
Never Married	38 (3.1)	22 (5.5)	3 (0.7)	13 (3.2)
Residence				
City	384 (31.7)	133(33.3)	174 (43.3)	77 (18.7)
Town	330 (27.2)	126(31.5)	80 (19.9)	124 (30.2)
Rural Area	499 (41.1)	141(35.3)	148 (36.8)	210 (51.1)
Religion				
Christianity	933 (76.9)	341(85.3)	329 (81.8)	263 (64.0)
Islam	276 (22.8)	59 (14.8)	73 (18.2)	144 (35.0)
Traditional	4 (0.3)	-	-	4 (0.9)
Level of Education				
No formal education	93 (7.7)	3 (0.8)	5 (1.2)	85 (20.8)
Primary Education	207 (17.1)	48 (12.0)	66 (16.4)	93 (22.7)
Secondary Education	572 (47.2)	211 (52.8)	219 (54.5)	142 (34.7)
Tertiary Education	339 (28.0)	138 (34.5)	112 (27.9)	89 (21.8)
Levels of Income				
No income	338 (28.5)	62 (15.5)	53 (13.3)	223 (57.5)
Below 20,000	693(58.4)	261(65.4)	294(73.5)	138 (35.6)
Above 20,000	156 (13.1)	76 (19.0)	53 (13.3)	27 (7.0)
Own a mobile phone				
Yes	1081(89.1)	379(94.8)	376(93.5)	326(79.3)
No	132(10.9)	21(5.3)	26(6.5)	85(20.7)
Watch Television regularly				
Yes	1111 (91.6)	386 (96.5)	383 (95.3)	342 (83.2)
No	102 (8.4)	14 (3.5)	19 (4.7)	69 (16.8)
Own a bank account				
Yes	602(49.6)	238(59.5)	225(56.0)	139 (33.8)
No	611(50.4)	162(40.5)	177(44.0)	272 (66.2)
Use the internet				
Yes	338 (27.9)	141 (35.3)	116 (28.9)	81 (19.7)
No	875 (72.1)	259 (64.8)	286 (71.1)	330 (80.3)
Socioeconomic Status				
Low	201 (16.9)	17 (8.5)	34 (16.9)	150 (74.6)
Middle	611 (51.5)	216 (35.4)	240 (39.3)	155 (25.4)
High	374 (31.5)	166 (44.4)	126 (33.7)	82 (21.9)

### Use of maternal healthcare services

The analyses indicate that most participants (94.8%) visited health facilities to receive antenatal care services during their index pregnancy. Of the 63 women that did not utilise antenatal care services, 55.6% were from Nasarawa State. Skilled practitioners with varying levels of qualifications attended 84.8% of births across the study areas. Close to one-third of births in Nasarawa State were delivered without the presence of a skilled attendant. The majority of births in Ondo State (85.5%) and Ekiti State (76.5%) took place in skilled birth facilities compared to just over half in Nasarawa State (58.2%). Births in Faith-Based Attendants (FBAs) were common in Ekiti (19.0%) and Ondo (9.2%) States but rare in Nasarawa State.

### Factors associated with facility-based childbirth

The analysis reveals that place of residence, educational level and socioeconomic status were associated with facility-based childbirth in Ekiti State, whereas only educational level was significantly associated with facility-based childbirth in Ondo State (Table 2). Availability of health facilities in the community of residence, age, place of residence, educational level and socioeconomic status were significantly associated with facility-based childbirth in Nasarawa State.

**Table 2:** Chi-square statistics showing variables associated with use of facility-based childbirth

Variables	Ekiti	Ondo	Nasarawa
Proportion of births in SBF	306 (76.5)	344 (85.6)	239 (58.2)
Age Groups			
20 and below	7 (46.4)	13 (100)	17 (41.5)
21-25	46 (74.2)	56 (88.9)	59 (52.2)
26-30	94 (75.2)	106 (87.6)	77 (63.1)
31-35	78 (82.1)	86 (83.5)	55 (70.5)
36-40	57 (76.0)	60 (85.7)	24 (54.5)
40 and above	23 (85.2)	22 (71.0)	6 (54.5)
*p*-value	0.06	0.12	0.03
Place of residence			
City	87 (65.4)	145 (83.3)	66 (85.7)
Town	96 (76.2)	69 (86.3)	100 (80.0)
Rural	123 (87.2)	129 (87.8)	73 (34.9)
*p*-value	<0.001	0.52	<0.001
Level of Education			
No formal education	3 (100.0)	5 (100.0)	24 (28.2)
Primary Education	36 (75.0)	51 (77.3)	34 (36.3)
Secondary Education	151 (71.6)	181 (84.4)	97 (68.3)
Higher Education	116 (84.1)	103 (92.0)	82 (92.1)
*p*-value	0.04	0.04	<0.001
Socioeconomic status			
Low socioeconomic status	11 (64.7)	25 (73.5)	41 (27.3)
Middle income	153 (70.8)	203 (84.9)	111 (71.6)
High Socioeconomic status	141 (84.9)	113 (89.7)	75 (91.5)
*p*-value	0.003	0.056	<0.001
Availability of health facilities in community			
Yes	306 (76.5)	344 (85.6)	198 (80.5)
No	0(0.0)	0(0.0)	41 (24.8)
*p*-value	N/A	N/A	<0.001

Key: *N/A* means not available, *SBF* means skilled birth facilities

### Who benefitted from user-fee removal?

Having adjusted for participants that reported user fees and what such payments were for, the overall proportion of beneficiaries of user-fee removal was 33.6% (see Table 3). This proportion varies by state and place of residence. The proportion of beneficiaries was highest in Ondo State (52.0%) and lowest in Nasarawa State (20.0%). The proportion of beneficiaries of user-fee removal policy was highest in urban areas (35.9%), women belonging to the middle income category (38.3%), women who gave birth in primary health centres (63.1%) and among women who resided in communities where there was availability of health facilities (37.2%).

**Table 3:** Beneficiaries of free maternal healthcare according to state, place of residence, socioeconomic status and place of birth

Variable	All *n* (%)	Ekiti State	Ondo State	Nasarawa State
Beneficiaries of free health	407 (33.6)	116 (29.0)	209 (52.0)	82 (20.0)
Place of residence				
City	138 (35.9)	28 (21.1)	80 (46.0)	30 (39.0)
Town	115 (34.7)	38 (30.2)	52 (65.0)	25 (20.0)
Rural	154 (30.9)	50 (35.5)	77 (52.0)	27 (12.9)
*p*-value	0.255	0.030	0.019	<0.001
Socioeconomic status				
Low socioeconomic status	36 (17.9)	3 (17.6)	15 (44.1)	18 (12.0)
Middle income	234 (38.3)	68 (31.5)	128 (53.3)	38 (24.5)
High Socioeconomic status	130 (34.8)	45 (27.1)	65 (51.6)	20 (24.4)
*p*-value	<0.001	0.369	0.599	0.011
Place of birth				
Home/Mission	0 (0)	0 (0.0)	0 (0.0)	0 (0.0)
Private	0 (0)	0 (0.0)	0 (0.0)	0 (0.0)
Tertiary health centres	95 (44.8)	0 (0.0)	70 (69.3)	25 (35.2)
Secondary health centres	76(52.4)	2 (5.0)	60 (89.6)	14 (36.8)
Primary health centres	236 (63.1)	114 (63.7)	79 (66.9)	43 (55.8)
*p*-value	<0.001	<0.001	<0.001	<0.001
Availability of health facility in community				
Yes	390 (37.2)	116 (29.0)	209 (52.0)	65 (26.4)
No	17 (10.3)	-	-	17 (10.3)
*p*-value	<0.001			<0.001

In Ekiti State, a higher proportion of women residing in rural areas (35.5%) and towns (30.2%) compared to cities (21.1%) benefited from user-fee removal. Similarly, in Ondo State, a slightly higher proportion of women residing in the rural areas (52.0%) and towns (65.0%) benefited from user-fee removal than those in cities (46.0%).

Contrastingly, only 12.9% of women in rural areas benefitted from user-fee removal compared to 39.0% of women residing in cities. Only 12% of women belonging to the lowest socioeconomic status compared to 24% of women in high socioeconomic status benefited from user-fee removal in Nasarawa State.

## Discussion

**Fig. 1:**
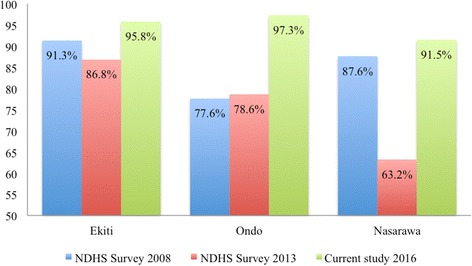
Trends in Antenatal care utilisation in Ondo, Ekiti, and Nasarawa states from 2008 to 2016

**Fig. 2:**
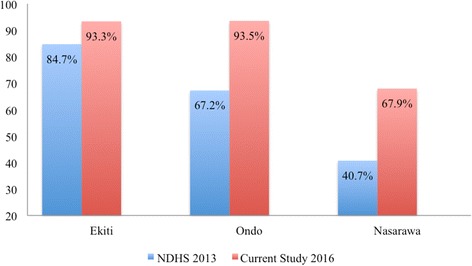
Trends in Births attended by skilled birth attendants in Ondo, Ekiti, and Nasarawa states from 2013 to 2016

This study examined the use of maternal healthcare services in the context of free maternal healthcare, and profiled the beneficiaries of free maternal healthcare in two main regions of Nigeria. The findings of this study suggest improved use of maternal health services compared to the 2013 and 2008 NDHS findings. In Ekiti State, the use of antenatal care services and proportion of births assisted by skilled birth attendants increased by nine points compared to the NDHS 2013 results (Figs. 1 and 2).

**Fig. 3:**
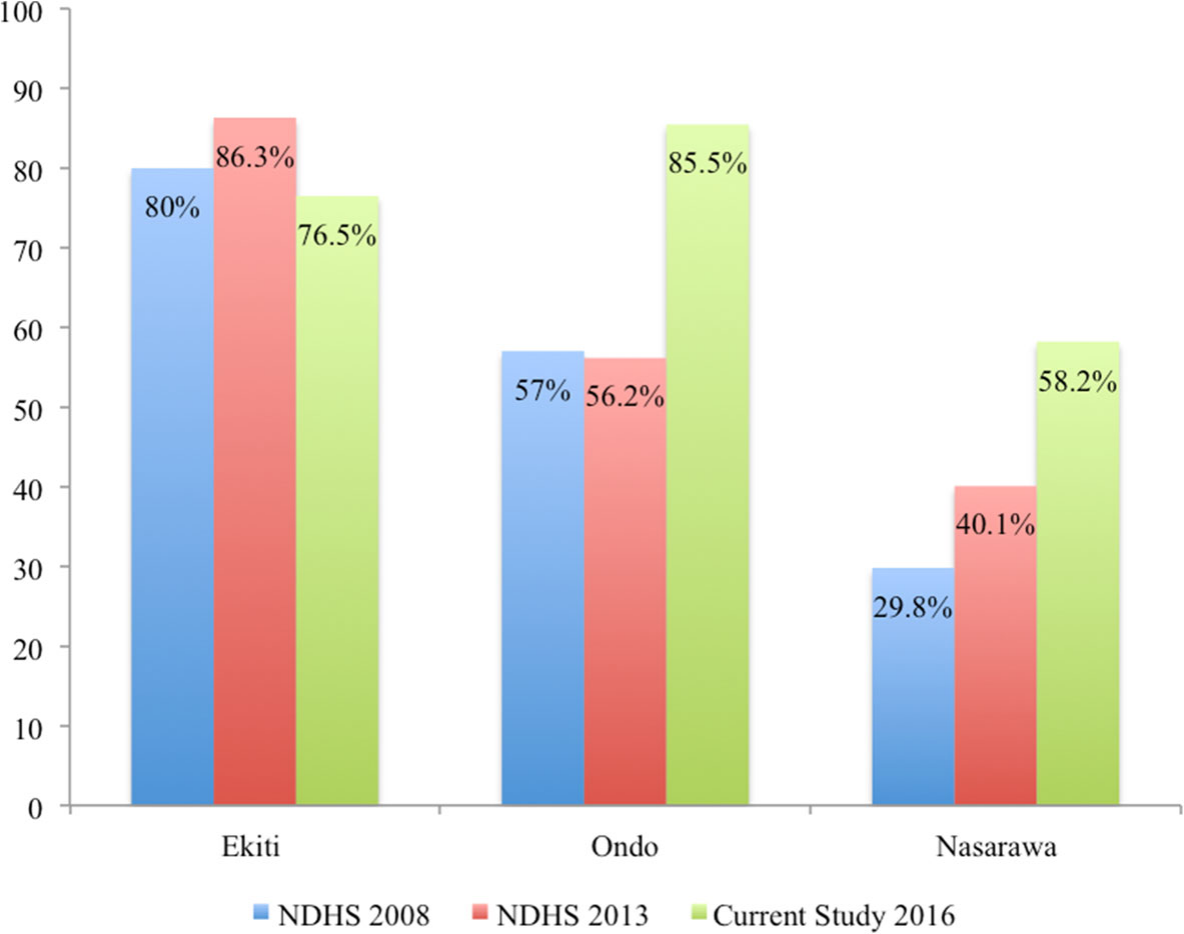
Trends in Births that took place in health facilities in Ondo, Ekiti, and Nasarawa states from 2008 to 2016

In Ondo State, the proportion of women utilising antenatal care services increased by 19 points. The proportion of births assisted by skilled birth attendants and births that took place in health facilities increased by 27 points (Fig. 3).

In Nasarawa State, the rate of utilisation of antenatal care increased by 28 points compared to the 2013 NDHS result. The proportion of births assisted by skilled birth attendants increased by 27 points, while the proportion of births that took place in skilled birth facilities increased by 18 points. Previous studies had linked improvement in the use of maternal healthcare services to the removal of user fees for maternal health [5–8]. However, it is important to note that the overall increase in the use of maternal healthcare services in the present study may not be entirely due to the removal of user fees policy. In Ondo State, for instance, the removal of user fees was complemented by massive health system strengthening and provisioning of new health facilities in underserved communities. Thus, it is unsurprising that the rate of increase in the use of maternal health services was highest in Ondo State. Overall the study findings present two different scenarios of implementation of user-fee removal for maternal healthcare services. In the South West region, where there is wide coverage of health facilities, removal of user fees seemed an effective strategy for improving the use of maternal healthcare services. However, in North Central Nigeria—where there is limited coverage of health facilities—removal of user fees seemed an ineffective approach to ensure universal access to maternal healthcare services.

Although the use of maternal healthcare services has significantly improved, women of the lowest socioeconomic status were still the least likely to receive antenatal care, give birth in skilled birth facilities and be assisted by skilled birth attendants. However, while the inequality in the use of maternal healthcare services appears to be disappearing in Southwestern Nigeria, it appears to be widening in North Central Nigeria despite the introduction of the free maternal healthcare policy. The persistent inequality despite the implementation of free maternal healthcare was reported by previous studies in many sub-Saharan Africa countries [9, 28–30]. A plausible explanation for the persistence of inequality in the use of maternal healthcare services in Southwestern Nigeria is the use of faith-based facilities; perhaps this was due to the poor quality of services offered under free maternal healthcare. It might even be associated with the fact that in Nigeria’s predominantly Christian southern region, childbirth is believed in many quarters to trigger complications which can only be overcome through spiritual interventions— and where best to find a ‘fitting’ combination of faith and healthcare than in a faith-based health facility?

The finding that the use of skilled birth facilities is common in rural areas of Ekiti State compared to urban areas is surprising. However, it suggests that quality issues such as prolonged waiting times in urban areas might be the reason why some women preferred faith-based facilities in spite of the high proportion of skilled birth facilities in urban areas of Ekiti State.

In contrast, persistent inequality in the use of maternal healthcare services in North Central Nigeria can be attributed to limited coverage of free maternal healthcare intervention, unavailability of health facilities, and urban bias in the allocation of health facilities and maternal health interventions. There was more coverage of free maternal healthcare in Southwestern Nigeria—a region with relatively better maternal health outcomes— than in North Central Nigeria. There was more coverage of free maternal healthcare in the rural areas of Southwestern Nigeria but coverage was city-biased in North Central, a region with poor maternal health outcomes. Furthermore, women of low socioeconomic status were the least likely to benefit from free maternal healthcare services in North Central Nigeria whereas this was not the case in Southwestern Nigeria.

The difference in the use of maternal health services in North Central and Southwestern Nigeria despite the implementation of free maternal healthcare explains why maternal health outcomes continue to be poor in settings where free maternal healthcare is introduced. Our findings challenge the assertion that the poor maternal health outcomes of poor women residing in settings where free maternal health initiatives were implemented was due only to poor quality of care, unofficial charges, lack of transportation and distance. Our findings show that the coverage of free maternal healthcare was not universal; thus, the poor women residing in underserved communities were the least likely to benefit from the user-fee removal for maternal health policy. This finding is in agreement with the findings El-Khoury et al. [17] and McKinnon et al. [10] findings but in contrast to the findings of De Allegri et al. [19].

The main reason for low use of maternal healthcare services in settings where free maternal healthcare was introduced as found in this study was non-availability of maternal healthcare services. In parts of the study settings where there were no health facilities, most women gave birth at home and were unaware of free child delivery in government hospitals. The findings further emphasise the importance of improving the availability of health facilities. Making child delivery free could be an important strategy to increase the use of maternal healthcare services in settings where there is availability of services. However, introduction of free maternal healthcare in settings where building health facilities is needed, such as the case of North Central Nigeria, is a policy mismatch.

To improve maternal health, there is a need for context-specific interventions. Need-based analysis is essential, and mapping of ‘hotspot’ areas requiring specific intervention should be a prerequisite for allocating funds for interventions. Currently, this appears to be lacking in both regions of Nigeria, and would be crucial if Nigeria is to achieve the Sustainable Development Goal of reducing the ratio of maternal deaths to 70 deaths per 100,000 live births by 2030. One study has advocated geospatial mapping of hotspot areas, where women mostly give birth at home, as a key strategy to improving the use of maternal health services [15]. Findings of the present study does emphasise this approach to improving the use of essential maternal healthcare services and preventing maternal deaths.

### Study limitation

One limitation to be considered in interpreting the results presented in this article is the potential for recall or memory bias as the data are from cross-sectional household surveys that retrospectively collect information about births within the past 5 years. There is potential for differential recall, especially on whether the childbirth was totally free. However, to determine beneficiaries of user–fee removal, participants were asked to report any out-of-pocket spending during childbirth and what specifically such spending was meant for.

## Conclusion

The findings of this study suggest that user-fee removal for maternal health services might be an important strategy for increasing use of essential maternal health services as demonstrated in Southwestern Nigeria. However, it is an ineffective strategy for addressing poor utilisation of maternal health services in underserved communities. In communities where home births were prevalent, the common denominator was non-availability of health facilities. Addressing inequality in the use of maternal health services would entail need based-assessment and prioritising the underserved communities in allocating interventions.
